# Surveillance of the first cases of COVID-19 in Sergipe using a prospective spatiotemporal analysis: the spatial dispersion and its public health implications

**DOI:** 10.1590/0037-8682-0287-2020

**Published:** 2020-06-01

**Authors:** Lucas Almeida Andrade, Dharliton Soares Gomes, Marco Aurélio de Oliveira Góes, Mércia Simone Feitosa de Souza, Daniela Cabral Pizzi Teixeira, Caíque Jordan Nunes Ribeiro, José Antônio Barreto Alves, Karina Conceição Gomes Machado de Araújo, Allan Dantas dos Santos

**Affiliations:** 1 Universidade Federal de Sergipe, Programa de Pós-Graduação em Enfermagem, Aracaju, SE, Brasil.; 2 Universidade Federal de Sergipe, Programa de Pós-Graduação em Biologia Parasitária, Aracaju, SE, Brasil.; 3 Universidade Federal de Sergipe, Departamento de Medicina, Aracaju, SE, Brasil.; 4 Secretaria Estadual de Saúde de Sergipe, Diretoria de Vigilância em Saúde, Aracaju, SE, Brasil.; 5 Instituto Federal de Sergipe, São Cristóvão, SE, Brasil.; 6 Universidade Federal de Sergipe, Programa de Pós-Graduação em Ciências da Saúde, Aracaju, SE, Brasil.

**Keywords:** COVID-19, Spatial analysis, Space-time clusters, Pandemic, Disease surveillance

## Abstract

**INTRODUCTION:**

Coronavirus disease 2019 (COVID-19) has become a global public health emergency with lethality ranging from 1% to 5%. This study aimed to identify active high-risk transmission clusters of COVID-19 in Sergipe.

**METHODS:**

We performed a prospective space-time analysis using confirmed cases of COVID-19 during the first 7 weeks of the outbreak in Sergipe.

**RESULTS:**

The prospective space-time statistic detected "active" and emerging spatio-temporal clusters comprising six municipalities in the south-central region of the state.

**CONCLUSIONS:**

The Geographic Information System (GIS) associated with spatio-temporal scan statistics can provide timely support for surveillance and assist in decision-making.

Coronavirus disease 2019 (COVID-19), caused by severe acute respiratory syndrome coronavirus 2 (SARS-CoV-2), stands out as one of the greatest public health challenges worldwide. This disease is characterized by a respiratory syndrome, ranging from mild upper respiratory disease to severe interstitial pneumonia and acute respiratory distress syndrome^1^.

The initial outbreak occurred in December 2019 in Wuhan, China, but on March 11, 2020, the World Health Organization (WHO) declared COVID-19 a global pandemic^1^. Due to the epidemiological dynamics and rapid geographical expansion of COVID-19, several measures to contain and mitigate the disease were implemented to flatten the contamination curve and prevent the collapse of national health systems^2^. In addition to the burden of assistance, surveillance systems have been under pressure to deal with the need to update the epidemiological situation, almost in real time. Most disease records are updated at least annually. When a health problem occurs in the territory, the epidemiological surveillance system must be able to quickly identify a new cluster of cases, regardless of their location and size, in order to determine the spatial patterns of disease occurrence.

Studies of space-time patterns can help to elucidate the mechanisms of disease spread in the population and identify factors associated with heterogeneous geographic distribution. Prospective space-time analysis is extremely useful for monitoring outbreaks, as it allows the detection of active, emerging clusters and the relative risk for each affected site during the epidemic^3^. This study aimed to identify active high-risk transmission clusters of COVID-19 in Sergipe.

An observational study with spatial analysis techniques was carried out, including all confirmed cases of COVID-19 in Sergipe and its capital, Aracaju, from March 12 to April 30, 2020, whose units of analysis were the 75 municipalities of Sergipe and the 39 neighborhoods of Aracaju. We collected data and the spatial locations of the residence of patients diagnosed with COVID-19 notified to the Department of Health of Sergipe (SDH/SE).

Sergipe (Figure 1A) is the smallest Brazilian state (21,918,454 km^2^) and located in the Northeast region of Brazil. It is divided into 75 municipalities and has a population of approximately 2,242,937. The municipality of Aracaju (Figure 1B), the state capital, occupies an area of 181.90 km², has 39 neighborhoods, and an estimated population of 623,766^4^.

**FIGURE 1:**
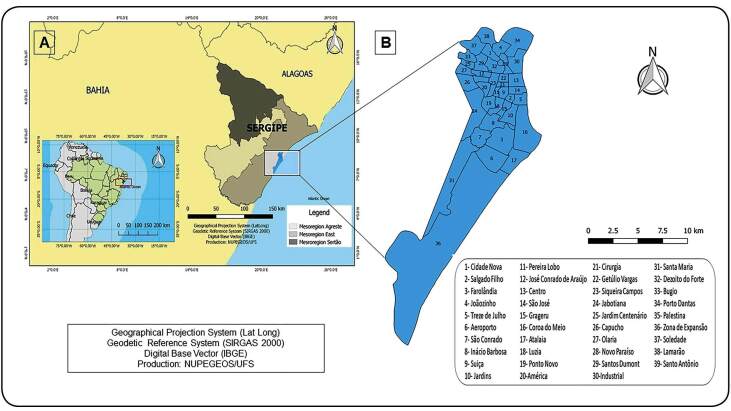
Study area.

Diagnostic tests for confirmation of COVID-19 using quantitative reverse transcription polymerase chain reaction (RT-qPCR) were performed at the Central Public Health Laboratory (Lacen). Lacen tests all symptomatic patients with influenza-like illness (ILI) at hospitals and sentinel units. It is important to note that during the period there were changes in the sample collection criteria for the SARS-CoV-2 RT-qPCR test. Initially, tests were performed for suspected cases coming from transmission areas, but after the decree of community transmission, the indication was restricted to cases that required hospitalization, or health workers.

The addresses of the confirmed cases of COVID-19 were georeferenced and points were marked from the capture of the latitude and longitude coordinates provided by Google Maps^5^. Subsequently, the non-parametric Kernel intensity test^6^ was applied. Through statistical smoothing, a density surface was generated for the visual detection of hot spots, indicating agglomeration in a spatial distribution and continuous surface from the georeferenced data. The amount of smoothing, that is, the width of the influence radius was defined as 3,000 m, as this value generated an adequate representation of the distribution of cases.

The prospective space-time scan statistic was performed to identify high-risk spatio-temporal clusters for transmission of COVID-19 using the discrete Poisson probability distribution model^3^. This analysis allowed the evaluation of potential clusters that were still occurring at the end of the study period. Those that were still occurring, that is, active, were considered active space-time clusters (present)^7^. Clusters that did not present a statistically significant relative risk were disregarded.

In this model, the number of cases in each location follows the Poisson distribution, having been chosen because the base population reflects a certain mass risk. The cases are then included as part of the population count. Under the null hypothesis, the expected number of cases in each area is proportional to the size of its population, or the person-years in that area. The Poisson discrete model requires counting cases and populations for a set of data locations^3^ ^,^ ^7^.

In the model we assume the COVID-19 cases follow a Poisson distribution according to the population of the geographic region. The null hypothesis H0 states that the model reflects a constant risk with an intensity μ, proportional to the population at risk. The alternative hypothesis AH states that the number of COVID-19 cases observed exceeds the expected number of cases derived from the null model (high risk)^3^. 

The expected number of COVID-19 cases (μ) under the null hypothesis H0 is derived as in Equation 1^3^:

$$\mu =p^{*}\;C/P$$ (1)

with p being the population in i; C the total number of COVID-19 cases in the region; and P the total estimated population in the region. Note that the model assumes that the population is static in each location at each time period.

A model was built with the following conditions: aggregation time of one day, without overlapping clusters, circular clusters, maximum size of the spatial cluster of 50% of the population at risk, and maximum size of the temporal cluster of 50% of the period of study. The most likely and secondary clusters were detected using the log likelihood ratio (LLR) test and represented through maps. The relative risks (RR) of the occurrence of COVID-19 were calculated for each cluster in relation to its neighbors. Results with p-values < 0.05 using 999 Monte Carlo simulations were considered significant^8^. 

Maps were made using QGis 3.4.11 (Open Source Geospatial Foundation, Beaverton, Oregon, OR, USA) and TerraView 4.2.2 (National Institute for Space Research, INPE, São José dos Campos, SP, Brazil). SaTScan™ 9.6 (Harvard Medical School, Boston and Information Management Service Inc., Silver Spring, MD, USA) was used for space-time scanning analysis.

Informed consent was not required as this study was solely based on publicly available secondary anonymous data, with no possibility of identification of individuals. Ethical review board approval was therefore not necessary. The researchers guaranteed the confidentiality and anonymity of all data, including personal information.

During the study period, 2,512 tests for the diagnosis of COVID-19 were performed in residents of Sergipe. A total of 453 (18.03%) cases were confirmed, of which 3 (0.66%) cases were excluded because the municipality of residence was not located in Sergipe or there was no available information regarding the place of residence.

The first cases of the disease were confirmed on March 14, 2020 in residents of Aracaju city, Atalaia neighborhood, south of the capital. The mean age of patients was 43 ± 18.9 years. They were predominantly female (54.70%), aged 20 to 59 years old (80.12%), and residents of the capital (65.56%). 

All cases of COVID-19 confirmed in the period were georeferenced and their spatial analysis is represented in Figure 2. Kernel analysis identified clusters of greater density (hot spots) of cases in the metropolitan region (Aracaju, Barra dos Coqueiros, and Nossa Senhora do Socorro e São Cristóvão) (Figure 2A) showing dispersion of the virus throughout Sergipe's territory. Other possible risk areas can be seen in the central region of the municipality of Aracaju formed by the Jardins, Grageru, Ponto Novo, Luzia, Salgado Filho, Inácio Barbosa, São José, and 13 de Julho e Suíça neighborhoods (Figure 2B). 

Figure 2C demonstrates the statistically significant emerging spatio-temporal clusters of COVID-19 between April 26 and April 30, 2020. The cluster is found in south-central Sergipe and includes 6 municipalities (Table 1) with an RR of 23.69 (p < 0.0001).

In Aracaju (Figure 2D), one statistically significant space-time cluster was observed. The cluster is formed by 19 neighborhoods (Atalaia, América, Cirurgia, Centro, Coroa do Meio, Farolândia, Grageru, Inácio Barbosa, Jabotiana, Jardins, José Conrado de Araújo, Luzia, Pereira Lobo, Ponto Novo, Salgado Filho, São José, Suíça, Siqueira Campos, and Treze de Julho), centrally located in the capital, showing an RR of 12.59 (p < 0.0001) (Table 1).

**FIGURE 2:**
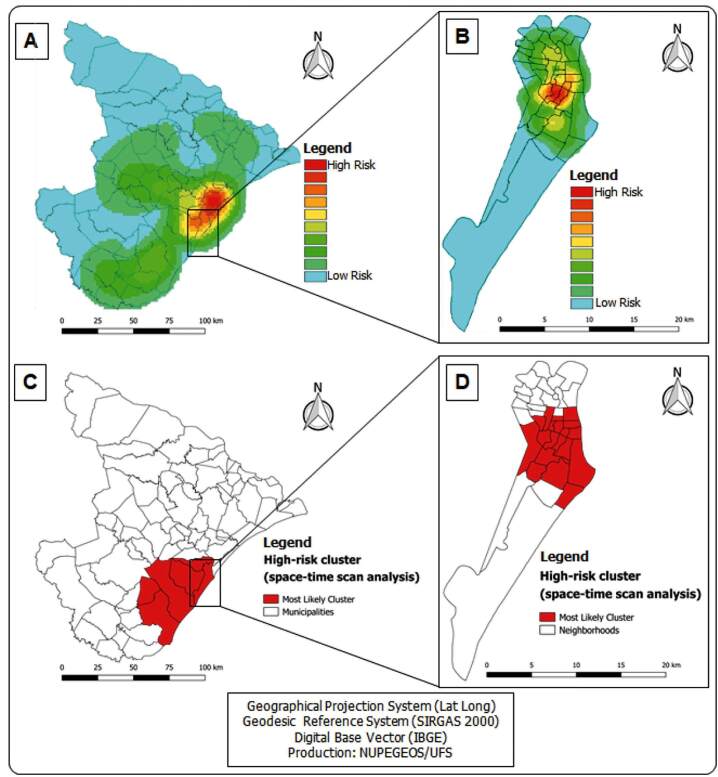
Spatial and space-time analysis of COVID-19, Sergipe, Brazil. (**A)** Kernel density map of confirmed cases of COVID-19 in Sergipe. (**B)** Kernel density map of confirmed cases of COVID-19 in Aracaju. **C)** Spatio-temporal scanning analysis in Sergipe. **D)** Spatio-temporal scanning analysis in Aracaju.

**TABLE 1:** Space-time clusters of COVID-19 from Mar 12th-April 30th, 2020 in the state Sergipe and Aracaju.

	Cluster	Duration (days)	Local	Expected	Observed	RR	LLR	p-value
Sergipe	1	Apr 26th - Apr 30th	Aracaju, Estância, Itaporanga D´Ajuda,	21.64	246	23.69	445.97	<0.0000001
			Salgado São Cristóvão,					
			Nossa Senhora do Socorro					
Aracaju								
	1	Apr 24th - Apr 30th	Atalaia, América, Cirurgia, Centro, Coroa do Meio, Farolândia,	21.5	152	12.59	200.6	<0.0000001
			Grageru, Inácio Barbosa, Jabotiana, Jardins,					
			José Conrado de Araújo, Luzia, Pereira Lobo, Ponto Novo,					
			Salgado Filho, São José, Suiça, Siqueira Campos, Treze de Julho					

**RR:** relative risk for the cluster compared with the rest of the region; **LLR:** likelihood ratio. The expected number of cases in each area is proportional to the size of its population in that area.

The results of this research propose space-time dissemination patterns of COVID-19 in Sergipe and its capital, Aracaju. To the best of our knowledge, this is the first study that uses prospective space-time scan statistics to map active clusters of the disease in our state. These findings may be useful for planning public health policy to deal with the epidemic. Similar studies, using technological devices aimed at understanding the spatial dynamics of COVID-19, have also been carried out in China, the country in which the first infections were reported^9^. 

Although it is a relatively low lethality disease, COVID-19 is characterized by its ease of dissemination and consequent saturation of health systems. In view of the international public health emergency, after the confirmation of the first five cases in the state, the state government issued the 40,560 decree (https://www.se.gov.br/uploads/download/midia/9/a3621f0448801b738cc5cce794263b49.pdf) to regulate COVID-19 pandemic measures. It is important to highlight that epidemiological surveillance control actions focusing on the monitoring of ILI in the state of Sergipe were initiated in the month prior to confirmation of the first cases, when negative results were obtained for all 54 patients with symptoms suggestive of SARS-CoV-2 that were tested.

The spread of the disease followed the patterns of other states. Initially, the cases occurred predominantly in the capital, especially in neighborhoods with higher income, as they were possibly related to infection whilst in other countries or contact with travelers. However, from the consolidation of community transmission, the disease spread to the south, forming an important space-time cluster in the central-southern region of the state. It is important to highlight that commercial activity and intercity mobility in these municipalities is intense, such as in Estância, the city that has the second highest number of confirmed cases to date.

The analysis of sociodemographic characteristics shows greater involvement of adults with a predominance of the 20- to 59-year-old age group, corroborating a South Korean investigation that also reported that most of the affected individuals were between 20- and 59 years old^10^. The average age of our cases, 43 years, is close to that reported by studies conducted in India (40.3 years)^11^. Individuals of all ages are generally susceptible to SARS-CoV-2^12^; however, studies have shown that the disease is more severe in the elderly, especially those who have pre-existing clinical conditions such as heart disease and diabetes mellitus^13^. This suggests that a weakened immune system might facilitate the evolution of the viral infection^14^. As for children, the presenting symptoms are relatively mild^12^ ^,^ ^14^. 

Regarding sex, there was a predominance of female individuals, corroborating an investigation that also found a higher percentage of women affected^10^. However, some studies have reported a preponderance of men^11^. 

In this context, remote sensing, big data spatial technologies, and the GIS have played important roles. Through these, it is possible to make space-time predictions about the speed of transmission and the scale of the epidemic, as well as to map the dynamics of the supply and demand of health resources in the territory^15^. In this study, use of the GIS allowed risk mapping in territories based on statistical associations between different types of data and knowledge about the disease distribution, enabling control programs to be more consistent with local realities. 

Faced with an emerging and rapidly spreading disease such as COVID-19, techniques that analyze space-time data support decision-making processes essential for disease surveillance in the geographical space. At-risk areas can also be identified to prioritize the allocation of health resources, and to implement interventions that facilitate the monitoring and control of cases^7^. 

COVID-19 is a growing global public health problem. Specifically in Sergipe, the problems posed are mainly due to the potential for dissemination, adaptability to new environments, universal susceptibility, and the possibility of extensive epidemics and occurrence of severe cases with respiratory problems. Thus, the emergence of any public health problem in an indene environment should never be neglected.

The fight against COVID-19 requires comprehensive policies and interventions that involve various sectors of society. Despite the difficulties related to socioeconomic factors, the health sector in this country needs urgent investment for the implementation of virologic epidemiological surveillance actions, especially at times of heightened risk to public health. 

The perplexity regarding the worldwide spread of COVID-19 and its impact in Brazil were sufficient for the Ministry of Health and the WHO to declare a public health emergency. This situation solicited an intense mobilization of resources and articulation between states and municipalities to combat the spread of the virus. 

Our study has some limitations. The provision of some information on the notification forms was optional. This can hinder the collection of necessary information, which can negatively affect epidemiological research and lead to distorted conclusions and inadequate public policy planning.

As the pandemic expands to other regions, new data can be added to the prospective space-time scan statistics to monitor active clusters and identify areas that are not at excessive risk from COVID-19^8^. Despite the relevance of the findings, it is necessary to consider the heterogeneity in the transmission between different regions, since these vary according to actions, routines, health service structure, and surveillance. Thus, future studies will be necessary to understand the impact of the disease in different territories. 
